# Longitudinal evaluation of a pilot e-portfolio-based supervision programme for final year medical students: views of students, supervisors and new graduates

**DOI:** 10.1186/s12909-017-0981-5

**Published:** 2017-08-22

**Authors:** Gillian H. S. Vance, Bryan Burford, Ethan Shapiro, Richard Price

**Affiliations:** School of Medical Education, Newcastle University, Level 2, Ridley building 1, Newcastle upon Tyne, UK

**Keywords:** E-portfolio, Medical student, Transition, Foundation Programme, Postgraduate medical education

## Abstract

**Background:**

Little is known about how best to implement portfolio-based learning in medical school. We evaluated the introduction of a formative e-portfolio-based supervision pilot for final year medical students by seeking views of students, supervisors and graduates on use and educational effects.

**Methods:**

Students and supervisors were surveyed by questionnaire, with free text comments invited. Interviews were held with new graduates in their first Foundation Programme placement.

**Results:**

Most students used the e-portfolio (54%) and met with their supervisor (62%) ‘once or twice’ only. Students had more negative views: 22% agreed that the pilot was beneficial, while most supervisors thought that e-portfolio (72%) and supervision (86%) were a ‘good idea’. More students reported supervision meetings benefited learning (49%) and professional development (55%) than the e-portfolio did (16%; 28%). Only 47% of students felt ‘prepared’ for future educational processes, though graduates noted benefits for navigating and understanding e-portfolio building and supervision. Factors limiting engagement reflected ‘burden’, while supervision meetings and early experience of postgraduate processes offered educational value.

**Conclusion:**

Final year students have negative attitudes to a formative e-portfolio, though benefits for easing the educational transition are recognised by graduates. Measures to minimize time, repetition and redundancy of processes may encourage use. Engagement is influenced by the supervisor relationship and educational value may be best achieved by supporting supervisors to develop strategies to facilitate, and motivate self-directed learning processes in undergraduates.

**Electronic supplementary material:**

The online version of this article (doi:10.1186/s12909-017-0981-5) contains supplementary material, which is available to authorized users.

## Background

Portfolios are increasingly used in healthcare education to support learning, personal and professional development and assessment. As a tool for students to record and reflect on clinical experiences, they may enhance knowledge, integration of theory with practice and encourage lifelong learning skills, including self-awareness and identification of learning needs [1]. However, whilst popular amongst educators, portfolio use has had mixed responses from learners: significant issues cited by graduate doctors have been the burden and bureaucracy of the process [2, 3]. Medical students have similarly raised concern about the time-consuming workload of a portfolio [4] and the purpose and benefit of reflective writing [5]. There is some evidence that an electronic (e-) format may increase student motivation, with greater time spent in portfolio preparation [6]. However, despite the ubiquity of technological access, no benefits of an online portfolio for medical student satisfaction or portfolio quality have been demonstrated [7].

As the sustainability and educational benefits of an e-portfolio-based learning programme are dependent on user engagement, it is essential to gather the views of both students and supervisors on the acceptability and value of the process [8]. There is limited literature around user perspectives on portfolios in undergraduate medical programmes in general, and further understanding of local contextual factors may help to ensure successful introduction [9].

An important unanswered question is how e-portfolio use by undergraduates affects their transition to postgraduate educational practice. Medical graduates in the UK undertake a two-year Foundation Programme in which progression is based on portfolio-based assessment. Authors have suggested that introduction to portfolio use during medical school may promote more positive attitudes among graduates through greater familiarity with processes [9] and increased confidence in portfolio building [10], both of which may improve educational engagement and focus on learning. However, despite these proposed benefits, empirical data on what effects an undergraduate programme may have on the experience of educational transition are lacking.

Hence, this study sought to evaluate the implementation of a formative personal development e-portfolio and dedicated supervision sessions for final year medical students at Newcastle University, UK. The study addressed three research questions:

1. To what extent do final year medical students and supervisors engage with a pilot e-portfolio-based programme?
2. What factors are associated with use and acceptability of the pilot e-portfolio by final year medical students?
3. What are the perceived educational effects of e-portfolio use during final year studies and through the transition to Foundation Programme practice?

## Methods

### Study design & setting

The evaluation study was carried out to support a pilot programme of e-portfolio-based supervision for final year medical students. The goals of the programme were three-fold: firstly, to support students in their academic progress; secondly, to encourage development of life-long learning skills and thirdly, to help students negotiate the transition to graduate educational practice. The study took place in a regional medical school where students are assigned to one of four areas (Base Units) across North East England and North Cumbria, and may have placements at different hospitals within that area.

### Participants and recruitment

All final year students on the undergraduate medical programme (2015–16, *n* = 355) were invited to take part in the programme, which ran through the course of the academic year. All were given access to the Undergraduate Medical e-Portfolio (UMeP), which is an electronic portfolio developed by a National Health Service (NHS) portfolio provider in partnership with a number of medical schools in the UK [9]. This e-portfolio has content and format based on that used by most UK new medical graduates (Foundation Programme trainees), but it was stressed that their use of it was optional. All students were invited to take part in the study whether they had used the e-portfolio or not.

### Use of e-portfolio and supervision

Students and supervisors were introduced to the goals and expectations of the programme through oral presentations and online materials. They were asked to devise a personal development plan (PDP) and upload evidence to demonstrate attainment of objectives within their plan. Some functions of the e-portfolio (example, supervisor meeting forms or requesting an assessment) could be used at the student’s discretion, while student versions of other assessment forms (example, Supervised Learning Events, multi-source feedback) were not included in the programme as the initiative was being piloted alongside established curricular processes. Students were each allocated a supervisor in the same Base Unit (*n* = 176), and encouraged to have at least three meetings throughout the year. All participants had access to support from the programme lead (RP) throughout the year.

### Evaluation questionnaire

An online questionnaire was sent to all students and supervisors at the end of the programme. This was designed in-house in order to gather data on use within the context of this programme and perceptions of its educational value. Questions were developed by the study team and refined to ensure clarity and local relevance following feedback from piloting with a number of students and clinical teachers. Questions used Likert scales with textual anchors (5-point scales ranging from ‘strongly agree’ to ‘strongly disagree’ or ‘very easy’ to ‘very difficult’). Anonymised free text responses about the portfolio-supervision process were also invited (supplied as Additional file 1; see References).

### Interviews

All graduates were contacted when they had begun Foundation year one (F1) and invited to take part in a follow-up telephone interview. Six volunteers (3 male) were interviewed in the final month of their first Foundation Programme placement. These semi-structured interviews were conducted by a non-medical researcher (ES) to minimise any barriers around disclosure. Interviews explored participants' experience of using the undergraduate e-portfolio and their perceptions of the effects of that experience having moved on to the Foundation Programme, where use of an e-portfolio is compulsory.

### Analysis

Questionnaire frequency data are reported, with responses recoded to simplify opinions where appropriate: for example, ‘strongly agree’ and ‘agree’ were aggregated, as were ‘strongly disagree’ and ‘disagree’. Differences in the frequencies of categorical data between student and supervisor groups were compared using chi-square tests in R (R Core Team [2017]; https://www.R-project.org/). A value of *p* < 0.05 was considered significant.

An inductive thematic analysis was conducted on qualitative data gathered through survey free text and interviews (treating the data as a single corpus), using methods described by Braun and Clarke [11]. Data was read and re-read by individual researchers before a coding structure was agreed by the team. Coding was then carried out at the level of sentences to paragraphs. Codes were collapsed and modified in order to identify themes in the data.

## Results

Questionnaires were received from 182 of 355 (51%) students and 125 of 176 (71%) supervisors. These response rates are good for this type of electronic survey, and while respondent bias cannot be excluded, representation of half of the student cohort provides some reassurance that a range of opinions has been gained.

### Use of e-portfolio

Of student respondents, most had used their e-portfolio just ‘once or twice’ (54% of 176 answering that question), with 33% using it 3–5 times, and 3% more than five times. Ten percent had not used the e-portfolio at all. At least 45% of the student cohort (159/355) had therefore used the e-portfolio to some extent. However, that more than half of these were not *regular* users (95/159, 60%) may be of some concern (Table 1).

**Table 1:** Reported frequency of e-portfolio use – students (responses *n* = 176)

Frequency	Response
Not at all	17 (10%)
Once or twice	95 (54%)
3–5 times	59 (33%)
More than 5 times	5 (3%)

Students indicated the frequency of e-portfolio use. Around half reported occasional use only (‘once or twice’) during the duration of the pilot programme (September to April)

Meetings with supervisors reflected these figures. Table 2 shows that the majority of students (62%) and supervisors (75%) reported meeting once or twice over the year, with just a small number indicating that they had never met (students 5%; supervisors 7%). However, only 33% of student respondents had met with their supervisor 3 or more times (as recommended).

**Table 2:** Reported frequency of supervision meetings

Frequency	Students (*n* = 177 responses)	Supervisors (*n* = 98)
Never	9 (5%)	7 (7%)
Once	64 (36%)	74 (75%)
Twice	46 (26%)	
Three times	32 (18%)	17 (18%)
More than 3 times	26 (15%)	

Students and supervisors indicated the frequency of supervision meetings. Most reported meeting ‘once or twice’ during the duration of the pilot programme (September to April)

### Overall opinions – Acceptability

Students were generally negative about the pilot overall, with only 22% (39/175) agreeing that it was ‘beneficial’ and 9% (16/176) that it had ‘met their expectations’. Conversely, supervisors were more positive with 72% (69/96) agreeing that the e-portfolio, and 86% (83/96) that supervision meetings were a ‘good idea’ for all final year students, though many reported that arranging meetings had been difficult (47%, 46/97) and that they would have liked more meetings (56%, 55/98).

Responses on the ease of e-portfolio use were mixed, with 20% of students finding it ‘difficult’ (35/176) and 34% ‘easy’ (59/176).

### Educational effects

Only 16% (29/177) of students agreed that using the e-portfolio had been beneficial to their learning, and fewer still (11%, 20/177) regarded it as having value for reflection.

Supervision meetings were regarded positively by more students (49%, 87/176, identifying some benefit for learning), although views on discussing reflections with supervisors were mixed: of 176 answering the question, 40% agreed and 31% disagreed that this opportunity was ‘enjoyable, satisfying and worthwhile’.

Conversely, supervisors were more positive and many felt that the pilot would benefit both students’ learning (52%, 58/111) and their professional development (57%, 64/112). They frequently regarded supervision meetings as ‘useful’ (61%, 57/93), allowing development of rapport with the student (57%, 54/94). While the PDP was regarded as helpful in guiding discussion (60%, 56/93), only a minority of supervisors (24%, 23/95) noted that the student had ‘made good use of the e-portfolio’ and, in keeping with students, relatively few supervisors associated e-portfolio use with development of reflective skills (27%, 26/95).

Table 3 illustrates the differing views of students and supervisors. Supervisors more often rated positively (‘agree’) questions about learning and professional development. There was a highly significant association between a positive response (‘agree’) and respondent group for items concerning learning outcomes (chi-square = 47.12, *p* < 0.0001) and professional development (chi-square = 27.99, *p* < 0.0001). Student items were: ‘Using the ePortfolio in final year has been beneficial to my learning’ and ‘Using the ePortfolio in final year has been beneficial to my professional development’. Supervisor items were: ‘I think that the pilot scheme will benefit the learning goals of final year medical students’ and ‘I think that the pilot scheme will benefit the professional development of final year medical students’.

**Table 3:** Distributions of student and supervisor responses to questions regarding learning outcomes and professional development

	Learning outcomes		Professional development	
	Disagree	Agree	Disagree	Agree
Student	104 (78%)	29 (22%)	76 (60%)	50 (40%)
Supervisor	25 (30%)	58 (70%)	18 (22%)	64 (78%)

Supervisors more often rated positively (‘agree’) questionnaire items about learning & professional development

### Anticipated benefits for transition to foundation Programme

Supervisors were also generally positive about future benefits: most (66%, 75/113) felt that the programme would ‘support transition of medical students to Foundation year 1’, whether as preparation for future ‘use of e-portfolio’ (69%, 77/112) or supervision processes (77%, 83/112). While many students also agreed that using the e-portfolio (53%, 96/180) or having supervision meetings (66.5%, 117/176) were ‘good practice for the future’, still, 15% (26/176) reported feeling ‘not prepared to make the most of Foundation supervision and e-portfolio processes’ compared with 47% (83/176) who were.

### Qualitative analysis

Our research questions considered use and acceptability of the e-portfolio. There were three factors that influenced these.

### Burden and redundancy

***‘I think my thing was that it was a burden I could have done without’. (Interview #5)***

Negative attitudes commonly related to a perceived ‘burden’ of the pilot experience. This burden often reflected a question of time, whether for e-portfolio building, or in the time-intensive nature of reflection. However it could also be in process, and in particular travelling to meet a supervisor if they were based at a different hospital, suggesting that this was not a problem with the pilot programme per se, but rather the specific organisational logistics.

‘My supervisor was not based at the hospital that I was in and therefore meeting him required 90 minutes of travelling for a 5-10 minute meeting which I didn't think was a useful way of spending my time’. (Student survey).

Also, to many students the e-portfolio appeared to duplicate their existing log book and was seen as additional workload that did not appear to align with current processes.

‘It seemed like something that was just bolted on because we had all of our log book outcomes and then we also had the supervisor/e-portfolio so it seemed like the two were trying to do very similar things, but from a different perspective’. (Interview #3)

This problem was inevitable since the pilot was running concurrently with the course log book, and so may not reflect feelings should the programme be fully implemented. Nonetheless, these ‘extra’ activities had to be balanced against the pressure to pass final examinations, and while many recognised potential *future* benefit, the activities were not seen as a priority in the *current* context of clinical sessions and looming exams.

‘The portfolio sort of went on that side of I need them for real life, but right now it’s not about real life, it’s about passing finals. It kind of went on the backburner’. (Interview #1)

### Clarity of purpose

A further factor limiting engagement arose from perceptions of a lack of clear purpose of the e-portfolio. A number of respondents simply felt that they did not understand what was expected of them, and despite an introduction session and supporting materials, students and supervisors were often unsure about how best to navigate the e-portfolio and what functions they could use. This could be a particular interface or user experience issue related to the specific e-portfolio platform, but questions of scale seem inherent to the amount of information contained in the portfolio.

‘Other people have struggled with both supervisor and medical students looking at the e-portfolio and going ‘we’re not quite sure what we need to fill in’, because it was a vast website with lots of forms that could be filled in’. (Interview #5)

To some students, the e-portfolio was seen as a'tick-box', and as an optional programme without summative assessment to help define purpose, both students and supervisors recognised that it was under-utilised. However, an advantage of the e-portfolio was noted in its providing grist for the supervision meetings, with one interviewee reporting that the supervision meeting was enhanced by, or even dependent on having an e-portfolio to discuss.

‘I think the most helpful thing for me was the supervisor meetings so I definitely think that…I don’t know if I would have them without the portfolio’. (Interview #1)

Ways to join up undergraduate and Foundation Programme processes were advocated. These included adopting a single online approach, which would be familiar to members of clinical teams with whom students come into contact. Moving completely online would also avoid the risk of losing paper log books.

‘I think it would be good to do away with the log books and have everything in your e-portfolio because certainly everyone clinical in hospitals is used to filling in tickets and e-portfolios, but you’re not going to lose it’. (Interview #3)

However, students were aware that online requests for assessment had the risk of not obtaining the required assessments from busy clinicians. They saw a paper-based system as being faster, easier and more reliable.

‘Getting paper log books signed off is much easier than getting the outcomes signed off online, just because it’s easier to put your book under someone’s nose and get it signed and to not have to chase people around and get it ticketed’. (Interview #5)

Going further than replicating the appearance and functionality of the postgraduate portfolio platform for undergraduate use, some suggested that sharing common portfolio content across the graduate transition could simplify and integrate processes.

‘Some of the things I had to do in fifth year I’m having to do again now. If I could retain those and not have to get things signed off this year I could be done and that would be quite useful, but I don’t know if that is actually possible’. (Interview #3)

### Timing within undergraduate programme

The third factor related to when portfolio-supervision was best implemented. Given perceptions of burden, some students and supervisors felt that the process would be of more relevance and use during ‘Student Selected Components’ [SSCs] (a timetabled placement in the fourth undergraduate year in this medical school, where the subject area is chosen by the student and provides opportunity to study the topic in greater depth), when they also had more time to commit to portfolio maintenance, and the portfolio would be of more practical use in helping to structure self-directed learning in these placements.

The e-portfolio was thought inappropriate for earlier years when students were adapting to the clinical environment or had too limited clinical exposure to allow evidence building. Some, however, also recognised that as an activity to support preparation for professional practice, there was particular relevance for implementation in the final year.

‘You don’t really record a lot of this knowledge in a very structured way and I think it would be good to be able to reflect the learning you’ve had in your SSCs and your e-portfolio’. (Interview #3)

### Educational value

The final research question considered the educational impact of the pilot, and specifically the effects of undergraduate participation on graduate preparedness for Foundation Programme processes.

Our survey data were generally negative in this regard and suggested that students viewed the e-portfolio as having limited educational benefit. However, the individual perspectives of new graduates gave insight into ways in which the process could have ‘added value’, and help ease the transition to postgraduate educational practice.

### Supervision

***‘That [supervision] was definitely the best portion of it for sure’. (Interview #2)***

The survey had revealed a clear view among students that supervision meetings provided most added value to participation in the pilot. This was reinforced by graduates who also highlighted that the attitude of the supervisor, and the relationship they established with the student, were central to this benefit.

In supervision meetings, students valued support and guidance, whether for academic, career or pastoral concerns. They noted the benefit of having a source of advice to address issues proactively, and the ‘safety’ of sharing concerns with someone who was not seen as part of the curriculum management team.

‘It was quite good talking to someone outside of the curriculum about…not that I particularly used it, but I did think it was helpful to talk to him about anything I was struggling with academically, but didn’t want to see someone off the course really, and he was very receptive so if I did have a problem I could have talked to him about that’. (Interview #5)

Supervisors included a number of teaching fellows in early postgraduate years, and this ‘near peer’ teaching-supervision relationship offered further advantages for feedback on student progress and giving the practical insight of a postgraduate e-portfolio user.

‘She talked about how she used her portfolio as she’s working towards becoming a GP so she told me what she put in it, which was a bit more advanced than what we were doing at the time’. (Interview #1)

Supervisor attitudes could influence how a student engaged with the programme and their perceptions of ‘burden’ or ‘benefit’. Supervisors with knowledge about the process, flexibility about meetings and enthusiasm encouraged student involvement. Similarly, how a supervisor responded to e-portfolio building could shape students’ views on its use.

‘He [supervisor] actually made me more engaged that I actually was going to [be] in the first place’. (Interview #5)

‘With my supervisor the process felt token/pretend and as a result was more of a nuisance to both of us’. (Student survey)

Conversely, supervisors described students’ varying attitudes towards, and engagement with the supervision process, which many found frustrating. However, the striking educational effects of differing supervisor attitudes and approaches may also indicate a need for faculty development at this level to ensure students receive a more equitable supervision experience.

### Reflective skills

Despite frequent negative survey scores, educational benefits for reflective and self-directed learning could be recognised by graduates, now that these were felt to be relevant to their stage of training.

The main expectation of students was that they would use the e-portfolio to draft a personal development plan and record evidence of attainment. This experience gave some students insight to self-directed learning and goal setting, though some found the task unduly prescriptive.

‘It has made me have to think about areas I particularly felt the need to improve on and forced me to be proactive about addressing them. Having an educational supervisor who was willing to engage with the process as equally as I was meant having someone to reflect on these areas with, and someone who could offer advice’. (Student survey)

‘I’m not sure how helpful it [the PDP] was because I think we were forced to come up with these fairly arbitrary goals’. (Interview #3)

Similarly a number of students viewed writing structured reflections as being ‘restrictive’, ‘repetitive’ and unnatural. However, some Foundation doctors acknowledged that they now found reflective practice to be challenging and that the undergraduate programme had given them valuable experience of this skill.

‘It definitely [helped] with the thing we don’t always like to do: reflections’. (Interview #4)

### ‘Trying it out’ eases the transition

***‘When you get to the real world things will change and you have to get used to that’. (Interview #3)***

Graduates shared ways in which the pilot could ease the transition to Foundation Programme educational requirements. These related to the advantages of ‘trying out’ the postgraduate processes. They highlighted the key significance of e-portfolio-based summative assessment in the Foundation Programme and this postgraduate ‘weight’ gave support to its use as an undergraduate. This was, however, couched in a scepticism about the value of the Foundation portfolio.

‘The e-portfolio is a very important part of F1. I don’t think it’s a very useful part, but it’s very important so the more exposure you get to that the better’. (Interview #3)

Graduates had real difficulties with the size and complexity of the Foundation e-portfolio, which took time to learn to navigate. They now saw a practical value of having had opportunity to negotiate these technical online functions as an undergraduate, which contrasted with the confident outlook of final year students - that they could be easily learned as an F1.

‘I spent hours on trying to work out where stuff is’. (Interview #1)

‘I think in hindsight I probably would have liked to have engaged with it more because it probably would have made my F1 year with the portfolio even easier’. (Interview #5)

‘The engagement with supervision and portfolio has not been in any way helpful to final year, and added an extra unnecessary stressor, and is something that can be easily picked up in Foundation years without prior preparation’. (Student survey).

This ‘trying out’ as a student also gave F1s confidence in planning and arranging supervision meetings, and how to better use supervision to its full potential.

‘You’re just expected to arrange the meetings, but you just take more in your stride because you’ve already done it last year’. (Interview #5)

‘I think sort of…it sounds daft actually, but talking to a stranger about your education and discussing issues you’re having, I think it’s something I personally am not good at, so in fifth year it was quite a benefit going into F1’. (Interview #5)

Specifically, those e-portfolio functions (PDP and reflections) that F1s had used as undergraduates were adopted most easily in Foundation, whereas other portfolio requirements, such as formative observation of clinical encounters (known as Supervised Learning Events [SLEs]), were unfamiliar and caused greater challenge.

‘I thought it was less of a struggle [PDP and reflection], and was able to do it right away whereas the other ones [SLEs] took me a couple of months just to get my head around’. (Interview #2)

Indeed, F1s and supervisors encouraged wider use of Foundation Programme assessment tools in the undergraduate e-portfolio with an eye on the benefits of knowing ‘how to do’ them in advance. Further, now in the real world, F1s felt that students should have more experience of trainee-led processes, which can often be frustrating to organize in practice. This was in contrast to the previous student preference for ‘easier’ paper-based assessment processes.

‘The lesson with that is so much is chasing and pestering and things like that, and I think the more people get used to that the better. I often see students giving up too easily because things aren’t put on a plate for them’. (Interview #4)

## Discussion

Any educational advantages of an e-portfolio can only be realised if users engage with the approach [12]. Hence, critical feedback from students and supervisors on how an e-portfolio is used, and what aspects of the e-portfolio and supervision process benefit learning, is essential for sustainable and effective implementation.

In our pilot programme we introduced a personal development e-portfolio, which had a format and content that closely reflected the one used by graduates in order to increase the salience of the tool. Further, each medical student had a dedicated supervisor to support development of self-directed learning skills. However, despite taking measures that have been previously reported to increase the success of portfolios [13], few of our students regarded the e-portfolio as beneficial for learning. The majority of supervisors did, however.

These undergraduate findings resonate with earlier work that has reported Foundation Programme doctors tend to regard portfolio learning negatively [3]. However, our follow up of students into their first postgraduate placement highlighted some practice benefits that could help remedy undergraduate educational approaches. The findings identified ways in which the undergraduate experience eased the transition to postgraduate training and these areas of educational gain could be exploited in a strategy for successful implementation of e-portfolio-based learning. Furthermore, our data reveal more detail on aspects of the programme content and process that can be modified in order to promote acceptability and value among undergraduates.

### Factors determining engagement

A key frustration about the process was a perception among students that the pilot introduced more workload in a busy final year schedule. While this workload included time for supervision meetings, the criticism was directed particularly at e-portfolio building. While our e-portfolio content requirements were deliberately kept lean, flexible and personal to encourage focus and relevance [8], still the common report was that e-portfolio building was an additional burden on top of current course requirements. This raises the question as to whether implementation of the process earlier in the undergraduate course might facilitate greater acceptance of the system by avoiding curricular change at a critical time in undergraduate studies. In a longitudinal study, medical student perspectives towards a portfolio assessment process became more positive over four years suggesting that it may take time for students to recognise the value of learning portfolios [4]. Hence, earlier introduction may allow the process to bed in ahead of the pressure of high-stakes examinations. Also, many of our students and supervisors echoed findings of earlier work, namely, that integration and alignment of e-portfolio functions with the curriculum was a necessary factor for success. Further, while for some the possibility of portfolio assessment raised concern, for many, this was a key motivator for more active use [5, 14].

The negative student views of the e-portfolio as a tool for reflection were striking and poor perceptions of using portfolios for reflective learning have been reported commonly in the medical education literature. However, it was also notable that the graduate participants could identify potential benefit of the undergraduate reflective process, with some admitting that they now found reflection to be difficult in Foundation practice and regretting that they had not made better use of the process at the time. Ross et al. [5] suggested that medicine may lack a culture of reflective learning, and in this work, only a minority of supervisors perceived e-portfolio use as a means for the student to develop reflective skills. The current data give us encouragement that greater engagement is achievable, but success may depend on how the reflective account is used in discussion. Further examination of what steps supervisors can take to more effectively review written reflections may help enhance the meaning and educational value of portfolio-based reflections.

Supervisors’ role in improving engagement is supported by theory. Beckers et al. [14] theorised about student engagement with an e-portfolio in terms of self-determination theory, which links motivation with feeling competent, autonomous and relevant to the task or other people in the group [15]. They proposed tutor use of scaffolding to help students adapt to task difficulty and encourage autonomy in the learning process. Other motivational theories also refer to the importance of making clear the relationship between the task and future practice. This ‘vertical integration’ is commonplace in clinical curricula, but does not have the same prominence in educational outcomes. Students need to make similar practice connections with their future professional activity if they are to engage with the purpose and value of the e-portfolio-based processes. To this end, while supervisors need to support students in making these links, there may be additional benefit from harnessing the experience of new graduates as near-peer supervisors. Their recent experiences of transition, and personal insight of *‘if I knew then what I know now’*, could lend further authenticity and relevance to the tasks.

### Educational benefits of e-portfolio-based supervision

Despite many negative perceptions, the pilot had a number of successes, specifically in relation to supervision. Supervision meetings were often viewed positively by both students and supervisors. The positive influence was dependent on the characteristics of the supervisor, and their establishing a meaningful relationship. The relationship was, in part, supported by a geographical match of student and supervisor that allowed easy access, but most influential were the attitudes and behaviours of the supervisor. Our data showed that students had varying experiences, and that those supervisors who were informed about and engaged themselves with the process and with individual students' needs could positively shape student perceptions, and actively encourage their involvement. The influences of supervisor attributes on student learning are well described [16] and previous studies have demonstrated how the form that supervision takes can determine the effectiveness of student self-directed learning skills [17], particularly among novice learners [18]. The current findings emphasise the added value of dedicated mentorship, even for experienced clinical students, and suggest that faculty training needs to highlight the impact of role modelling and how positive attitudes could be best transmitted to the students.

The other main area of benefit was in the practice of postgraduate educational processes. Students anticipated value in terms of understanding the terminology, layout and functionality of the e-portfolio that they would be using in F1, but the actual value was captured from F1s, who described how the previous experience of both e-portfolio building and supervision processes gave advantages for saving time in operating e-portfolio and adapting to the educational expectations and frustrations of the ‘real world’. They found this transition difficult and indeed many of our group of participants advocated greater exposure to postgraduate assessments and technical tools as medical students. However, time saved in the Foundation Programme needs to be weighed up against time needed in the undergraduate course. Furthermore, implementation needs to maintain a careful balance between facilitating *‘practical training’* for future graduates and promoting self-directed *learning* skills for professional practice. Nevertheless, future roll-out may be strengthened by measures that explicitly articulate the challenges of educational transition and how the programme can help address these areas.

## Limitations

Our data give a view from only one medical school and hence the findings may not be transferable to other organisations. Further, while there was an adequate response rate to the survey, we cannot exclude a response bias. Also, the small number of interviews conducted poses additional risk of bias, though we have mitigated this risk by considering wide-ranging free text responses in the analysis. Hence, though this was an exploratory study, there were a range of views represented and the challenges of adapting to postgraduate educational processes shared by these graduates are likely to be relevant to all doctors entering the Foundation Programme. Our findings therefore suggest practical ways of helping to modify medical students’ perceptions of e-portfolio-based learning that will inform other undergraduate medical programmes wishing to implement e-portfolio-based learning processes.

## Conclusion

This study has suggested that implementation of a personal development e-portfolio for final year medical students can ease the transition to professional practice by supporting navigation of postgraduate processes. The potential benefits of such ‘educational simulation’ could be harnessed by medical schools to demonstrate professional relevance and encourage student engagement with e-portfolio use. However, engagement also requires consideration of measures that help minimize time, repetition and redundancy of processes. Further, its educational value is best achieved by supporting supervisors to develop strategies to facilitate, and motivate self-directed learning processes in undergraduates.

## Additional file

Additional file 1: Evaluation questionnaires. Student and supervisor versions of questionnaire (cascaded online during evaluation study). (DOCX 31 kb)

## Abbreviations

e-portfolio: electronic portfolio
F1: Foundation year 1 doctor
NHS: National Health Service
PDP: Personal Development Plan
SLE: Supervised Learning Event
SSC: Student Selected Component
UMeP: Undergraduate Medical ePortfolio
