# Supplementary material for: Longitudinal evaluation of a pilot e-portfolio-based supervision programme for final year medical students: views of students, supervisors and new graduates
Source: BMC Med Educ. 2017 Aug 22;17:141. doi: 10.1186/s12909-017-0981-5 (PMC5567902; doi:10.1186/s12909-017-0981-5)
Supplement: Additional file 1: — Evaluation questionnaires. Student and supervisor versions of questionnaire (cascaded online during evaluation study). (DOCX 31 kb) [file 12909_2017_981_MOESM1_ESM.docx]

**E-portfolio and supervision in final year MBBS – Students’ Viewpoint**

*Dear student,*

*This questionnaire seeks your views about the final year e-portfolio and supervision system. The questionnaire aims to find out which aspects of the system you feel are beneficial, what aspects you enjoy and gather your thoughts on any future changes needed.*

*Thank you for completing this questionnaire. Your responses will help us to improve the e-portfolio/supervision process.*

***Please check one answer that matches best with your views on the following statements.***

*Current attitudes to the system*

1. **To what extent do you agree with the following statements? (1= Strongly disagree; 5= strongly agree).**

**Firstly, regarding the e-portfolio:**

|  | **1 (Strongly disagree)** | **2** | **3 (Neither agree nor disagree)** | **4** | **5 (Strongly agree)** |
| --- | --- | --- | --- | --- | --- |
| Using the e-Portfolio in final year has been beneficial to my **learning**. |  |  |  |  |  |
| Using the e-Portfolio in final year has been beneficial to my **professional development**. |  |  |  |  |  |
| Using the e-Portfolio in final year **provides good practice** for e-Portfolio use in the future. |  |  |  |  |  |
| Having a **reflective outlet** in the e-Portfolio in final year is enjoyable, satisfying & worthwhile. |  |  |  |  |  |

1. **Regarding the supervision/ group tutor process:**

**To what extent do you agree with the following statements? (1= Strongly disagree; 5= strongly agree).**

**Regarding the supervision/ group tutor process:**

|  | **1 (Strongly disagree)** | **2** | **3 (Neither agree nor disagree)** | **4** | **5 (Strongly agree)** |
| --- | --- | --- | --- | --- | --- |
| Meeting with my supervisor during final year has been beneficial to my **learning**. |  |  |  |  |  |
| Meeting with my supervisor during final year has been beneficial to my **professional development**. |  |  |  |  |  |
| Meeting with my supervisor during final year **provides good practice** for similar encounters in the future. |  |  |  |  |  |
| **Discussing reflections** on my experiences during final year with my supervisor is enjoyable, satisfying & worthwhile. |  |  |  |  |  |

*Use of the -ePortfolio and meeting with your supervisor*

1. While you have had access to the **e-Portfolio**, on approximately how many occasions have you used it?

**Not at all // Once or twice // 3-5 times // More than 5 times**

1. How many times have you met with your supervisor?

**Never // Once // Twice // 3 times // More than 3 times**

1. How easy did you find using the e-Portfolio?

**Very easy // Easy // Neither easy nor hard // Difficult // Very difficult**

*Your experience overall*

1. Overall, how would you rate the e-portfolio/supervision process?

| **1 (Not all beneficial; very much a nuisance).** | **2 (More of a nuisance than beneficial).** | **3 (Equally beneficial and a nuisance).** | **4 (More beneficial than a nuisance).** | **5 (Very beneficial; not at all a nuisance).** |
| --- | --- | --- | --- | --- |
|  |  |  |  |  |

1. To what extent has the process met your expectations?

| **1 (Not at all met my expectations)** | **2** | **3 (Met but not exceeded my expectations)** | **4** | **5 (Greatly exceeded my expectations)** |
| --- | --- | --- | --- | --- |
|  |  |  |  |  |

Please provide more detail to your answers above

|  |
| --- |

1. Overall how prepared do you feel to make the most of the Foundation supervision and e-portfolio process?

| **1 (Not at all prepared)** | **2** | **3 (Feeling equally prepared and not)** | **4 (Feeling prepared)** | **5 (Very prepared)** |
| --- | --- | --- | --- | --- |
|  |  |  |  |  |

What comments do you have on the following?

| **What has worked well in the UG supervision/portfolio process?**  **What has not worked so well?**  **What suggestions do you have for improvement?** |
| --- |

1. As a F1 doctor may we contact you to take part in a focus group to discuss your experiences in more detail?

**Yes // No //**

**E-portfolio and supervision in final year MBBS – Supervisors’/Tutors’ Viewpoint**

Dear colleague,

*This questionnaire seeks your views about the final year e-portfolio and supervision system, which you are helping to pilot. The questionnaire aims to find out which aspects of the system you feel are beneficial, what aspects you enjoy and gather your thoughts on any future changes needed.*

*Thank you for completing this questionnaire. Your responses will help us to improve the e-portfolio/supervision process.*

**Question 1: What is your specialty?**

| **Medical Speciality** | **Surgical Speciality** | **Psychiatry** | **General Practice** | **Other – please specify** |
| --- | --- | --- | --- | --- |

**The next few questions ask about your views on the undergraduate pilot:**

**Question 2: How much do you agree with the following statements?**

|  | **Strongly Agree** | **Agree** | **Undecided** | **Disagree** | **Strongly Disagree** |
| --- | --- | --- | --- | --- | --- |
| **I think that the pilot scheme will better prepare students for use of e-portfolio in Foundation Year 1** |  |  |  |  |  |
| **I think that the pilot scheme will better prepare students for supervision processes in Foundation Year 1** |  |  |  |  |  |
| **I think that the pilot scheme will support the transition of medical students to Foundation Year 1** |  |  |  |  |  |
| **I think that the pilot scheme will benefit the learning goals of final year medical students** |  |  |  |  |  |
| **I think that the pilot scheme will benefit the professional development of final year medical students** |  |  |  |  |  |

**The next few questions relate to your experience in the pilot.**

**Question 3: How much do you agree with the following statements?**

|  | **Strongly Agree** | **Agree** | **Undecided** | **Disagree** | **Strongly Disagree** |
| --- | --- | --- | --- | --- | --- |
| **My student has made good use of the e-portfolio** |  |  |  |  |  |
| **My student is developing reflective skills through using the e-portfolio** |  |  |  |  |  |
| **My student is developing reflective skills through the supervision process** |  |  |  |  |  |
| **Supervision meetings with my medical student are useful** |  |  |  |  |  |
| **I have been able to build a good rapport with my medical student through the supervision process.** |  |  |  |  |  |
| **Using the personal development plan in supervision meetings has been helpful to guide discussion** |  |  |  |  |  |

**Question 4. How many supervision meetings have you had?**

| **None** | **1-2** | **More than 2** |
| --- | --- | --- |

**Question 5: On average how long did meetings with your trainee last?**

| **Less than 30 minutes** | **30-45 minutes** | **Over 45 minutes** |
| --- | --- | --- |

**Question 6: How much do you agree or disagree with the following statements?**

|  | **Strongly Agree** | **Agree** | **Undecided** | **Disagree** | **Strongly Disagree** |
| --- | --- | --- | --- | --- | --- |
| **I would like to have more meetings with my medical student trainee** |  |  |  |  |  |
| **Finding time to meet with the medical student has been difficult** |  |  |  |  |  |

**Question 7: What aspects of the e-Portfolio/supervision process do you find MOST valuable?**

|  |
| --- |

**Question 8: What aspects of the e-Portfolio/supervision process do you find LEAST valuable?**

|  |
| --- |

And finally…

**Question 9: How much do you agree with the following statements?**

|  | **Strongly Agree** | **Agree** | **Undecided** | **Disagree** | **Strongly Disagree** |
| --- | --- | --- | --- | --- | --- |
| **I believe that the e-Portfolio is a good idea for all final year students** |  |  |  |  |  |
| **I believe that the supervision process is a good idea for all final year students** |  |  |  |  |  |
| **I would encourage other teaching/supervision staff to be an undergraduate supervisor** |  |  |  |  |  |

**Question 10: Have you any other comments about the undergraduate e-portfolio/supervision process - and how it might be improved – in the light your supervision experience with final year students.**
